# The effect of trust and its antecedents towards determining users’ behavioral intention with voice-based consumer electronic devices

**DOI:** 10.1016/j.heliyon.2022.e09271

**Published:** 2022-04-14

**Authors:** Debajyoti Pal, Pranab Roy, Chonlameth Arpnikanondt, Himanshu Thapliyal

**Affiliations:** a School of Information Technology, Innovative Cognitive Computing (IC2) Research Center, King Mongkut's University of Technology Thonburi, Bangkok 10140, Thailand; b School of VLSI Technology, Indian Institute of Engineering, Science and Technology, Shibpur 711103, India; c School of Information Technology, King Mongkut's University of Technology Thonburi, Bangkok 10140, Thailand; d Department of Electrical Engineering & Computer Science, University of Tennessee, Knoxville, TN 37996, USA

**Keywords:** Artificial intelligence, Consumer electronics, Intelligent social agent, Online survey, Privacy, Trust

## Abstract

Advances in artificial intelligence (AI) have ushered in a new era of consumer electronic (CE) devices: the voice-based CE devices (VCED's). A striking feature that separates these from other CE devices are their anthropomorphic capabilities. While current CE research has given a strong focus on improving various technical and security aspects of the VCED's, not much efforts have been given to explore their diffusion and acceptance in the society. However, if the CE community is to progress then there is an urgent need to view these systems from a sociotechnical perspective and take the user perceptions into account for further product development. In this work we propose a novel research framework by incorporating Human Computer Interaction (HCI) theories and Para Social Relationship Theory for exploring the effect of trust on the behavioral intention of users towards VCED's, keeping in mind their human-like attributes. Data is analyzed using a Structural Equation Modelling approach from 675 users of VCED devices from two Asian countries. Results show that the functional aspects of performance and effort expectancy, and social aspects of presence and cognition affect the trust factor. Privacy concerns do not affect trust. Overall, the results suggest that users treat VCED's as social objects employing social rules while interaction that indicates a dual nature of anthropomorphic systems. Suitable suggestions are provided for CE researchers for future research.

## 1 Introduction

Recently the use of voice-based consumer electronic devices (VCED) has been on the rise. These are very popular now and being used on a daily basis. There are a variety of such devices commercially available from smartphones, smart-speakers, tablets, to various other smart-home accessories like smart-plugs, smart-lamps, smart-locks, etc. However, recent works have shown that consumers use these devices for basic activities and are generally reluctant to carry out tasks like online shopping or financial transactions by using their voice [1]. Privacy and security provided by the VCED's is a major concern area for the consumers, and such concerns reflect a lower level of trust between humans and machines [2, 3]. In this aspect the consumer electronics (CE) community is doing an excellent work in terms of improving the security of the devices by introducing sophisticated encryption algorithms or other advanced cybersecurity principles [4, 5, 6]. A lot of focus is also being given to the privacy aspects of these VCED's, by incorporating the *privacy-by-design* philosophy [7, 8]. An equal stress is being given by the CE community to leverage the benefits of recent technologies like machine learning (ML), natural language processing (NLP), and artificial intelligence (AI) to improve various usability aspects of the VCED's [9, 10]. Therefore, the current research focus of CE researchers is based on these three pillars of security, privacy, and usability of the VCED's.

Unlike other CE devices, the VCED's have one unique aspect: their ability to build a relationship with their users due to the humanlike (anthropomorphic) features that they possess [11]. In fact, this has been a primary goal of CE researchers to make the devices more engaging, and as humanlike as possible. While this is a reality today due to the advancements made in NLP and AI, it opens up a new and less investigated research area of how humans build a relationship with these devices that affect trust, which in turn affect the behavioral intention. Such a trust aspect is an extremely important factor that needs to be investigated to ensure a proper diffusion of the VCED's in the mass market, however research on this aspect is scant [12, 13, 14]. Therefore, the present scenario demands investigating the human-machine relationships from a broader perspective involving the social aspects rather than the technical aspects only, which has traditionally been the focus and strength area of the CE researchers. Such a sociotechnical analysis of trust is very important as it helps giving insights to the technology adoption and use aspect, although very less research efforts are being given to explore this aspect in the CE context [14].

The central research theme of this work is “*how trust affects the behavioral intention of the users towards VCED's that possess anthropomorphic features?*” We believe that this is an important research problem for the CE community, since it will help researchers in this field to amalgamate the appropriate technical factors with the relevant social aspects that will lead to a greater customer engagement with these devices. To achieve this objective, we hypothesize three different dimensions of trust based on human-computer interaction (HCI) and information systems (IS) literatures. These three dimensions are the technical (functional) [15], hedonic [16], and the social attributes [17] of the VCED devices. We argue that the anthropomorphism and intelligence possessed by the VCED's present a unique context towards trust evaluation; an aspect that has seldom been investigated by extant literatures [18]. In fact some recent studies on AI-based service robots have not only identified trust to be an important factor related to their acceptance, but the level of anthropomorphism also affects trust [19, 20]. However, extant literatures on VCED's, as in [21, 22] have focused mainly on the technical aspects due to which there are limited studies to understand the role of trust as a challenge to the adoption process. Further, a few works do consider certain social aspects of the VCED's under the theoretical lens of theories like the Parasocial Relationship Theory (PSR) [23, 24]. Nevertheless, a comprehensive understanding of what affects the customers trust towards the VCED's has not yet been developed fully. Therefore, combining the technical and social approaches for creating a theory-based understanding for the adoption of the voice-based CE devices will be beneficial. By integrating the theoretical understandings of technology acceptance, the social aspects, and the anthropomorphic specialty of the VCED's, this work explores the functional, emotional, and social factors that influences the trust perceptions towards these devices. This work directly responds to the findings from very recent works in [19, 25] that calls for further exploring the consumers interaction with VCED's, especially the trust beliefs. By integrating concepts from the Unified Theory of Acceptance and Use of Technology model (UTAUT2), PSR together with VCED specific variables like perceived privacy risks, perceived humanness, and perceived intelligence we propose a theoretical model followed by its empirical validation that will help the CE community better understand the various trust antecedents, how it affects the behavioral intention, and what future research direction should be undertaken to enhance the man-machine relationships for ensuring a wide diffusion of the VCED's in the mass CE market.

## 2 Literature review

### 2.1 The functional aspect of VCED devices

VCED's are anthropomorphic by nature as they can emulate human-like traits by conversing with humans in various spoken languages. They are able to provide various types of personalized services due to their capability of striking human-like conversations. The AI attributes possessed by these devices make these unique and differentiate their acceptance scenario from other technology use-cases [26, 27, 28]. The first reason due to which humans interact with VCED's is due to their functional aspects. In this regard extant literatures have conveyed the importance of the functional attributes of technology. For e.g., perceived usefulness (PU), and perceived ease of use (PEOU) from the Technology Acceptance Model (TAM) are often found to be the key drivers of adopting any technology across a variety of research settings, including the VCED's [21, 22]. Some other studies have used related concepts of effort expectancy (EE) and performance expectancy (PE), adapted from UTAUT/UTAUT2 models [29, 30]. PE refers to the “*extent an individual feels that using a system is useful*”, while EE is defined as “*the level of easiness related to the system usage*” [31]. This functional aspect is highly relevant in the VCED usage scenario. Voice-based navigation, voice-based search, or even controlling the home equipments with voice are just some of the basic functionalities that these devices provide. This utilitarian aspect of VCED's has been established by extant literatures by using different theories like TAM, UTAUT, Value-based Adoption Model (VAM), etc [21, 22, 29, 30]. The second reason for interacting with the VCED devices is due to their hedonic capabilities [32, 33]. Their ability to play games, play a variety of music or even their ability to answer unknown questions creates an enjoyment level for the users. Extant studies have investigated this hedonic aspect of the VCED's and concluded that it is an equally important attribute along with the utilitarian aspect of these devices that shapes the consumer's perception. Yet, how both these dimensions drive the consumers trust is an unexplored issue. Although the effect of trust on reliance and resistance towards technology has been investigated, yet under the presence of anthropomorphism how trust is developed is not clear. While some of the other CE literatures have stressed on the importance of trust, especially from a security and privacy perspective, but how this trust affects behavioral intention is not clearly understood [3, 12, 13, 34].

### 2.2 The social aspect of VCED devices

The social aspect of VCED usage is related to the phenomenon by which humans apply social rules and expectations to computers or any digital devices, even though they are fully aware that such devices do not have any feelings, emotions, or motivations like the humans. In this scenario, people treat these devices as social actors, rather than just a medium, by assigning human traits (e.g., gender or ethnicity) and characteristics (e.g., reciprocity or dominance) to them [35]. Greater the human-like characteristics displayed by the devices, more is the effect of interpersonal social responses with the devices [35]. Extant research has shown that there are different types of such anthropomorphic cues like object shape (aesthetics), interactivity, and most importantly voice [36]. Hence, it is natural that the VCED's will show aspects of perceived humanness because they are able to get engaged in voice conversations with humans. Therefore, what type of bonds humans develop with these devices, and whether they consider them to be friends or foes is unknown [35, 37]. A few literatures have explored the social aspect arising due to the anthropomorphic features of the VCED devices in relation to level of consumer engagement, user's self-disclosure behavior and loyalty. These studies provide evidence that VCED's are “*socially present*” due to which users apply social responses when interacting with them [23, 35, 36, 38]. In Table 1 a summary is provided in relation to the dual aspects of functionality and sociability of the VCED devices.

**Table 1:** Result summary for extant literatures on VCED devices.

#	Study Focus	Factors	Theoretical Model	Trust Focus
[3]	Functional aspect	BP, EP, IA, TLA, OT, DMA	Self-proposed	-
[21]	Functional aspect	ATT, PU, PEOU, BI, ASU, SN, PBC, EE, PE, SI, FC, PRT, ENJ, PV	TAM + TRA + UTAUT + VAM	✓
[22]	Functional aspect	ATT, PU, PEOU, SN, ENG, LC, LOY	Modified TAM	×
[23]	Social aspect	TA, SA, PA, SPR, PR, SAT, CI	PSR	×
[26]	Functional aspect	PU, PEOU, ATT, BI	TAM	×
[29]	Functional aspect	EE, PE, SI, HM, PV, FC, PPR, PPC, PT, BI, ASU	UTAUT2	×
[30]	Functional aspect	EE, PE, SI, FC, PPR, BI	UTAUT	×
[32]	Both aspects	EE, PE, HM, SP, SA, PPR, BI	U&G	×
[33]	Both aspects	PU, PEOU, ENJ, HM	TAM + ISS	×
[35]	Social aspect	PSP, PF, SI, SD, SS	CASA	×
[36]	Social aspect	PH, PR, RM	PIT	×
[38]	Social aspect	SCI, ES	Self-proposed	×

**Note:** ATT (Attitude); PU (Perceived usefulness); PEOU (Perceived ease of use); BI (Behavioral intention); ASU (Actual system use); SN (Subjective norm); PBC (Perceived behavioral control); EE (Effort expectancy); PE (Performance expectancy); SI (Social influence); FC (Facilitating conditions); PT (Perceived technicality); ENJ (Enjoyment); PV (Perceived value), ENG (Engagement); LC (Localization); LOY (Loyalty); HM (Hedonic motivation); PPR (Privacy risk); PPC (Perceived privacy concern); PRT (Perceived trust); BP (Bystander privacy); EP (Environmental privacy); IA (identity assurance); TLA (Temporal & location assurance); OT (Openness & transparency); DMA (Data minimization assurance); TA (Task attraction); SA (Social attraction); PA (Physical attraction; SPR (Security/privacy risk); PR (Parasocial relationship); SAT (Satisfaction); CI (Continuance intention); PSP (Para-social presence); PF (Para-friendship); SI (Stickiness intention); SD (Self disclosure); SS (Social support); Perceived humanness; RM (Recommendation); SP (Social presence); SA (Social attraction); TAM (Technology acceptance model); TRA (Theory of reasoned action); VAM (Value-based adoption model); U&G (Uses and gratification theory); CASA (Computers and social actors); PIT (Parasocial interaction theory); SCI (Social identity); ES (Extended self); ISS (Information System Success).

### 2.3 The underlying theoretical framework

TAM is one of the fundamental theories for explaining technology adoption [39]. Its predictive capability has improved over time as researchers have used this model extensively in a variety of technology adoption contexts [40]. For example, Venkatesh et.al. created a short an alternative version of TAM and named it UTAUT, which was primarily focused in an organizational context [31]. However, with the popularity of digital media and various smart-devices, a newer version of UTAUT was proposed (named UTAUT2) having additional factors [41]. The newer model was also able to explain more variance in the usage of technology when compared to the original version. One aspect that differentiates UTAUT2 from UTAUT is that the former one considers the hedonic nature of an information system, while the later one does not. The functional aspect of the VCED devices involve both a utilitarian and hedonic attribute as evident from our literature review. Moreover, as seen from Table 1 different theories have been used for explaining the phenomenon of technology acceptance, however, using acceptance model like UTAUT2 is rare. Advancements in ML and AI techniques have radically changed the way people interact with the VCED's and now they are an integral part of any smart-home system. Therefore, using UTAUT2 as the theoretical backbone will help us in explaining this functional aspect of the CE devices.

Second, for considering the anthropomorphic features of these devices, and consequently their social aspect, we resort to the PSR theory [42]. Although this theory has its roots in an interpersonal context, it has been used for explaining human-machine relationships also [23, 36]. “*Interpersonal attraction*” is an important component of PSR, which suggests that the engagement level increases with an increase in attractiveness. The intelligence possessed by the VCED's together with their capability to strike natural language communications with the users, make them objects of social interest and social companionship. Therefore, PSR is ideally suited to explore the social aspect of the VCED devices. To the best of our knowledge, current adoption related studies on VCED devices have not taken such a dual sociotechnical approach for evaluating the trust antecedents. Using the unique theoretical combination of UTAUT2 and PSR will provide a new perspective to the current adoption scenario.

### 2.4 Efforts by the CE community & the research gap

From the above literature review the research gaps are evident from a CE perspective. First, traditionally the CE focus has been to improve the security of the various devices from a technical perspective. For example, authors in [43] have used a blockchain based approach to secure the data exchanges that occur between consumer IoT devices. SDN-based firewalls are being used to protect the user's IoT devices [4, 44]. Various ML and deep learning techniques are being embedded within the IoT devices and networks to cope with the security problems [45]. However, in a greater interconnected IoT environment as of today, how such security and privacy mechanisms translate to a good end-user experience and how the users trust such devices is an issue less explored by the community. As an instance authors in [13] investigated the consumer trust towards IoT technology and use of IoT products. However, their presented model is purely conceptual and does not focus on the trust aspect per se. Moreover, they consider general IoT devices, and not specifically the AI and anthropomorphic features of these devices that may influence the trust level. How ethics are important in an IoT scenario and its relation to social cues have been investigated by authors in [12]. Primarily they propose the concept of social affordances, and the importance of social network paradigm for the evolution of human society. An understanding of what affects trust, and its relationship with anthropomorphic technologies is however missing. Third, although stress is given to improve the AI and anthropomorphic features of the VCED devices using cutting edge ML, or NLP techniques, yet whether such improvements are translating to a trusted feeling among the users is not known. Security, privacy, and trust are hot topics of research among the CE community, yet whether such advancements are affecting the human characteristics and leading to a trusted environment, which further affects the behavioral intention towards using these devices i.e., their societal diffusion is an under-explored area by our community. Therefore, the main focus of this work is to advance the existing CE literature by understanding these sociotechnical aspects of trust and how it leads to the diffusion in the CE mass market and society.

## 3 Hypothesis development

### 3.1 Functional aspect and trust

**Figure 1:**
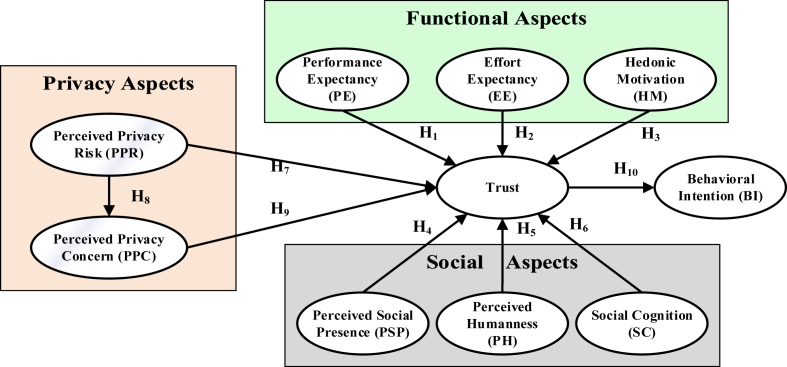
Proposed Research Model Investigating Trust and its Antecedents on Behavioral Intention in VCED Context.

Before developing the hypothesis, we present a working definition of trust for the current research context. Although trust can be conceptualized in a multitude of ways from a dispositional, institutional, or interpersonal perspective [46], for this work we used the interpersonal flavor. This is because voice-based systems have the capability of building relationships with their users that we previously discussed in the literature review section. The interpersonal flavor of trust tries to capture the expectations of the users with regards to the attributes or characteristics of the trustee, i.e., the VCED devices. Since in this work we explore the social, functional, and privacy aspects of VCED's, we define trust as “*the expectations of the users concerning the social, functional and privacy attributes of the trustee, i.e., the VCED devices as pre-conditions to consider their usage*”. The details of each of these aspects is discussed shortly. It is expected that the VCED devices will have characteristics that are beneficial for the users, and act in their interest. Figure 1 presents the research model.

In the VCED usage scenario the core functional elements of performance expectancy (PE) and effort expectancy (EE) are important and can act as a barrier to adoption if it does not match with the customers expectation levels [19, 25, 47]. Several studies have found out that these two aspects are important predictors of consumer's trust in an online setting [48]. The consumer's e-trust is also affected by the technical features of a system, such as the ease of navigation, the ease of searching information or even the quickness of response. These aspects have been considered in a variety of other AI-based application scenarios too. We dropped the habit construct of the original UTAUT2 model as one of the direct determinants because the VCED's are relatively new, and the user's need some time to develop a habitual behavior with a technology [29]. Similarly, the price value construct is also dropped since this study does not focus on the financial benefits/losses that arise due to technology use. As part of UTAUT2 the following hypotheses are proposed for the utilitarian aspect of the VCED devices:

H_1_: Performance expectancy positive influences user trust
H_2_: Effort expectancy positively influences user trust

Apart from the functional aspects, prior research has shown that the enjoyment (hedonic motivation) of using a technology also leads to its adoption [31, 32]. In certain scenarios related to mobile apps such hedonic motivations have been found to be stronger than the functional aspects [49]. The enjoyment associated with technology use also significantly influences the users trust towards technology [16, 50]. This is extremely relevant for the VCED devices as the consumer's interaction with such devices provide valuable benefits in terms of fun and enjoyment. Therefore, we propose:

H_3_: Hedonic motivation positively influences user trust

### 3.2 Social aspect and trust

When technology mimics human-like attributes, individuals apply social cues and treat digital devices as a social entity [35, 36]. Such a bondage and relationship development between man and machines go beyond the traditional factors like social influence or subjective norms that are common in HCI and IS literatures. Most often these factors refer to the social obligations or pressure that users form as a part of society towards technology use, however, the anthropomorphism aspect is related to social closeness between humans and machines that comes from within (intrinsic vs. extrinsic). Existing literatures on AI-based services adoption have revealed that these anthropomorphic design cues are indeed important, and help framing perceptions about these services [51, 52]. In this respect we propose three factors: perceived social presence (PSP), perceived humanness (PH), and social cognition (SC). From PSR theory, PSP can be defined as “*the extent to which the VCED devices make individuals feel as if they are in the presence of another social entity*”. The ability of these CE devices to communicate in natural language and indulge in engaging conversations makes them distinctly visible. Due to such human-like conversational flow the users may believe that the devices are really “*present*” just like another human and respond to them socially. Such social presence has been found to enhance consumer trust in technology, and a key factor towards its success [17, 53]. Based on this we propose:

H_4_: Perceived social presence positively influences user trust

When individuals anthropomorphize an object, they enter into a relationship with it, which triggers a trusting belief [25, 54, 55]. Once any technology is anthropomorphized, there is a feeling of connectedness towards the non-human agent. The users tend to develop closer relationships and trust with such devices. Such emotional exchanges are critical and an ongoing part of human-machine relationships that forms an important basis for bonds to be developed [54]. Hence,

H_5_: Perceived humanness positively influences user trust

Social cognition refers to “*how an individual process, store, and apply information about other people*” [56]. In this regard, competence is an important aspect that reflects the issues of intelligence, skill, and efficacy [56]. Since VCED devices use voice as the mode of communication (which is reserved for human-human communication), they might be perceived to be more sociable [57]. However, there is certain point beyond which this “*sociable aspect*” can lead to uneasiness [54]. Nevertheless, it can be expected that these AI powered devices will be reliable in terms of their functionality, and even be more competent and intelligent, when compared to other technologies. Thus, it is expected that interaction with the VCED devices will trigger the user's competence perception, resulting in inspiring trust. Therefore, it is hypothesized,

H_6_: Social cognition positively influences user trust

### 3.3 Perceived privacy and trust

Since neither UTAUT nor PSR considers the highly relevant issues of privacy that exists in human-machine interactions, we investigate two factors in this regard: perceived privacy risk (PPR), and perceived privacy concern (PPC). In the realm of VCED device usage, negative effects of privacy are well known [13, 32]. PPR is a subjective notion and refers to “*the opportunistic behavior of the service providers that lead to a loss on the part of consumer*”. Thus, this construct mainly portrays the fear that exists in user's mind with regards to their loss of confidential information [58]. The way tech giants collect and use information is debatable, especially because in most of the cases they are done without user's consent [29, 32]. The VCED devices are in some form related to these tech giants (either in terms of hardware or software) due to which the customers can experience high privacy risks. Greater such risks, lesser will be the trust. Therefore, we hypothesize:

H_7_: Perceived privacy risk negatively influences user trust

PPC on the other hand is related to the user's perception with regards to their privacy loss. The concepts of PPR and PPC are correlated and current research has also shown that PPR directly affects PPC [58]. This type of privacy concern is generated due to unauthorized collection of data, unconsented data usage, errors during data processing, or even leakage of personal information [3, 29]. Such concerns will negatively affect the benefits of using the VCED's along with the trust level. Thus, the notion of perceived privacy has a negative effect on trust, while trust has a positive influence on the adoption scenario [58]. Therefore, in the VCED context it becomes important to incorporate the privacy aspect in addition to the functional, hedonic, and social aspects that contribute to their behavioral intention. Thus, we propose:

H_8_: Perceived privacy risk positively influences perceived privacy concern
H_9_: Perceived privacy concern negatively influences user trust

### 3.4 Trust and usage intention

Trust is a very important issue that has shown to impact user's perception, as well as adoption of any technology [59]. It helps in overcoming the privacy risks [60]. Integrity, competence, and benevolence are some of the different aspects of trust that current literatures have considered [61]. However, the present context of VCED devices is unique, as they are capable of building relationships with the users, unlike other technologies. Therefore, the trust formation is examined from the viewpoint of the functional, social, and the privacy aspects. Nevertheless, higher trust levels should translate to a greater usage intention.

H_10_: Trust positively influences the behavioral intention

## 4 Methodology and data collection

A quantitative online survey technique is used for data collection and evaluating the research model. An informed consent was obtained from every participant taking part in the survey, and moreover no personal information like e-mail id's were recorded. The survey was conducted by using Google Forms for a duration of 2 months (January and February 2021). The survey was divided into two parts. The first part contained a set of 26 items corresponding to the various constructs used in the research model. All the items were adapted from previous studies Specifically, the UTAUT2 constructs (PE, EE, HM, and BI) were ported from [31]. For the social aspect, PSP was adapted from [32], PH from [37], and SC from [62]. For the privacy aspects PPR and PPC were adapted from [58, 63], and trust from [17, 63]. All the items were measured on a 5-point Likert scale ranging from 1 (strongly disagree) to 5 (strongly agree). In the second part of the survey general participant demographics were captured, for e.g., age, gender, education level, and usage experience with VCED devices. Before conducting the survey, a pilot test was carried out with 12 experts having knowledge with CE devices, and long-term experience of research in HCI and technology adoption. Although all the measurement items used in this study are based on established scales as mentioned above, yet the present context is new and unique. Consequently, we felt it is necessary to reframe the questions (if needed), due to which an initial pilot study was carried out. As mentioned, this pilot testing was done mainly to improve the readability of the questionnaire and based on the received feedback the final survey questionnaire was modified. For example, item numbers PE_3_, PSP_1_, PSP_3_, SC_2_, and PPC_4_ were re-worded based on the suggestions received. The final questionnaire details are presented in Table 2.

**Table 2:** Measurement items, reliability, and validity assessments.

Construct	Measures	Mean	Loadings	α value	CR	AVE
PE	PE_1_: I find VCED's to be useful in my daily life	3.58	0.947v	0.862	0.971	0.919
	PE_2_: Using VCED's increases my productivity	3.32	0.965			
	PE_3_: Using VCED's increases my chances of achieving tasks that are important to me	3.51	0.964			
EE	EE_1_: Learning how to use the VCED's is easy for me	3.95	0.938	0.820	0.935	0.885
	EE_2_: My interaction with the VCED's is clear and understandable	3.58	0.938			
	EE_3_: I find the VCED's easy to use	3.84	0.946			
HM	HM_1_: I find using VCED's to be fun and entertaining	3.82	0.926	0.851	0.941	0.888
	HM_2_: Using VCED's is enjoyable and exciting	3.85	0.958			
PSP	PSP_1_: When I interact with the VCED's I feel like I am dealing with a real person	3.80	0.854	0.847	0.904	0.758
	PSP_2_: When I interact with the VCED's I feel there is a touch of sociability	3.92	0.884			
	PSP_3_: When I interact with the VCED's I feel there is a sense of human sensitivity	3.67	0.873			
PH	PH_1_: Some I feel that the VCED's have real feelings	2.82	0.950	0.897	0.952	0.909
	PH_2_: I can imagine the VCED's to be real living beings	2.87	0.956			
SC	SC_1_: I think that the VCED's are intelligent	3.44	0.940	0.838	0.925	0.860
	SC_2_: I think that the VCED's are competent	3.47	0.914			
PPR	PPR_1_: I fear that VCED data could be given to unknown persons or companies without my consent	3.76	0.840	0.816	0.870	0.691
	PPR_2_: I fear that the personal data present in the VCED's may be misused	3.71	0.801			
	PPR_3_: I fear that the VCED data may be sold to third parties	3.82	0.850			
PPC	PPC_1_: I am concerned that the information I submit to the VCED's may be misused	3.36	0.842	0.849	0.889	0.667
	PPC_2_: I am concerned that my personal details stored on my VCED's could be stolen	3.34	0.820			
	PPC_3_: I am concerned that the VCED's collects too much information about me	3.35	0.814			
	PPC_4_: I fear submitting information to VCED's because what others might do with it	3.27	0.788			
Trust	TST_1_: I feel that the VCED's are trustworthy	2.99	0.894	0.908	0.901	0.819
	TST_2_: I believe in what my VCED tells me	3.01	0.916			
BI	BI_1_: It is likely that I will use the VCED's in the future	3.23	0.925	0.873	0.915	0.843
	BI_2_: I expect to continue using my VCED's in the future	3.51	0.911			

Data was collected from 700 respondents who took part in the survey. The online survey was conducted across two Asian countries (India and Thailand). However, 25 responses were unusable because they were incomplete, leaving the final sample size to 675. A mixture of purposive and convenience sampling technique was used for recruiting the participants. For this study we targeted young users who use VCED devices (belonging to the millennial generation). Instead of focusing on a specific device, we considered the popularly used ones like Amazon Alexa, Google Home, Siri, Cortana, and Bixby to improve the generalizability of the proposed research model and enable capturing the state-of-art related to the usage of these voice-based systems. The millennial segment was chosen because this is a young age group. The CE industry is giving special attention to this young generation as normally they are the early adopters of any new innovation and show a greater predisposition towards new technologies [64]. Therefore, we felt that this user group was most appropriate for the current study as most of them may have some familiarity with VCED devices. Additionally, in order to ensure that our target millennial group had sufficient familiarity with these devices, a screening question was used at the beginning of the survey. Only those who had at least 6 months of usage experience were allowed to continue with the main survey, while for all others they were not allowed to continue. Most of the participants were female (52%), belong to the age group of 26–33 years (76%), had either an undergraduate (43.4%) or graduate degree (31.6%), and between 1 – 2 years of experience in using VCED devices (49.4%).

Before conducting the Structural Equation Modelling (SEM) the data normality was checked in terms of the acceptable skewness and kurtosis levels. All the values were within the acceptable range of ±2 and ±3. Additionally, the Kolmogorov-Smirnov statistic was also found to be significant that suggests the collected data is fairly normally distributed. Additionally, any survey research is normally associated with bias. In order to keep the bias within acceptable limits a common method bias (CMB) was examined using a Harman's single factor analysis. The level of variance (36.4%) was found to be acceptable that is below the threshold of 50%. For analyzing the data, a Partial Least Squares method of SEM (PLS-SEM) is used. PLS-SEM is well suited for analyzing complex models (both reflective and formative), and also for both exploratory and confirmatory research types based on the total variance. There are several reasons for adopting a PLS-based approach in this work as mentioned next. First, in the research model that we proposed in this article, the objective was to estimate the different model parameters so that the explained variance of the endogenous constructs is maximized. On the contrary, in other SEM techniques like covariance-based approach (CB-SEM), the model parameters are estimated so that the discrepancy between the estimated and sample covariance matrices is minimized. Second, PLS-SEM is more generous towards normality distributions. Although, for the present case the data distribution is normal, PLS-SEM is suited for this type of data also. In fact, PLS approach can be used for both normal, and non-normal distributions. Third, our proposed research model has 10 constructs and 10 hypotheses. This makes it moderately complex, and in many scenarios such models (structural) do not converge. For CB-SEM this convergence is not guaranteed, however, PLS approach always converges. Finally, in our proposed research model we tried to explore new relationships starting from a hypothesized model that has reasonably good theoretical support (exploratory in a broad sense). Such scenarios are best suited for PLS-based approaches. However, when the focus is on exploring new relationships without having a hypothesized model (exploratory in a strict sense), CB-SEM is often preferred over PLS (which is not in the present case).

## 5 Data analysis and results

A two-stage procedure of data analysis is used by evaluating the measurement model first, followed by the structural model.

### 5.1 Measurement model evaluation

The measurement model is checked for reliability (composite and indicator) and validity (convergent and divergent) assessments. The composite reliability (CR) and average variance extracted (AVE) are examined for each construct (Table 2). The CR and AVE values are greater than 0.60 and 0.50 respectively, which are above the recommended threshold, and therefore the model does not show any composite reliability or convergent validity issues [65, 66]. Further, the Cronbach's Alpha values are calculated for each construct and found to be greater than the threshold value of 0.70 (Table 2). Therefore, it is established that the used scale has good convergence, reliability, and validity measures. The discriminant validity of the measurement model was assessed based on two criteria. First, we checked the Fornell Larcker criterion in terms of the square-root of AVE for each of the latent constructs (must be greater than the correlation with any other constructs) [66], and the Heterotrait-monotrait (HTMT) ratio of correlations test, wherein the HTMT statistics cannot exceed 0.85 [67]. Table 3 provides the inter-item correlation matrix. All the elements above the diagonal represent the HTMT statistics. Results show sufficient discriminant validity. Lastly, the overall measurement model is assessed by using a bootstrapped Standardized Root Mean Square Residual (SRMR) value, which is the most commonly used Goodness of Fit statistic in PLS-SEM based studies [68]. We obtained a SRMR value of 0.05 that is less than the threshold of 0.08, suggesting good model-fit [68].

**Table 3:** Inter-item correlation matrix, discriminant validity, and HTMT statistics.

Construct	PE	EE	HM	PSP	PH	SC	PPR	PPC	Trust	BI
PE	0.959	0.528	0.521	0.502	0.422	0.507	-0.117	-0.124	0.435	0.558
EE	0.574	0.941	0.459	0.494	0.478	0.454	-0.086	-0.128	0.501	0.613
HM	0.532	0.484	0.942	0.474	0.407	0.433	-0.115	-0.129	0.551	0.534
PSP	0.486	0.501	0.488	0.871	0.353	0.409	-0.117	-0.138	0.427	0.377
PH	0.437	0.492	0.429	0.389	0.953	0.364	-0.099	-0.137	0.198	0.367
SC	0.528	0.471	0.452	0.412	0.379	0.927	-0.102	-0.121	0.376	0.402
PPR	-0.129	-0.098	-0.143	-0.133	-0.118	-0.102	0.831	0.513	-0.209	-0.186
PPC	-0.136	-0.116	-0.156	-0.167	-0.143	-0.124	0.527	0.817	-0.126	-0.114
Trust	0.471	0.504	0.587	0.441	0.245	0.398	-0.212	-0.133	0.905	0.598
BI	0.577	0.618	0.552	0.386	0.368	0.401	-0.198	-0.129	0.613	0.918

**Note:** The diagonal elements are square-root of AVE, lower-diagonal elements are the correlation values, upper-diagonal elements are the HTMT statistics.

### 5.2 Structural model evaluation

In the second stage the structural model is evaluated for testing the proposed hypotheses by following a bootstrap approach. Bootstrapping is a resampling technique used to estimate the statistics of a population by independently sampling a dataset with replacement from the original sample The PLS algorithm was run using 5000 bootstrapping samples. The results of hypothesis testing are presented in Table 4. PE has a direct effect on trust (β=0.419,p=0.004), thereby H_1_ is supported. H_2_ is supported, meaning that EE positively influences trust (β=0.346,p<0.001). H_3_ is not supported, indicating the non-significant effect of HM on trust (β=−0.079,p=0.236). Among the social aspects, the effect of PSP on trust is confirmed (β=0.187,p=0.011), supporting H_4_. However, PH does not have any effect on trust (β=0.092,p=0.822), H_5_ not supported. Effect of SC on trust is significant (β=0.512,p<0.001), thereby supporting H_6_. Amongst the privacy related hypotheses, the effect of PPR on trust is significant (β=−0.565,p<0.001), supporting H_7._ The effect of PPR on PPC is also significant supporting H_8_ (β=0.626,p=0.002). However, the relation between PPC and trust (H_9_) is non-significant (β=−0.003,p=0.924). Finally, the relation between trust and BI is also found to be non-significant (β=0.032,p=0.431).

**Table 4:** Structural estimates and hypotheses testing.

Hypothesis #	Relationship	Standardized Weight (β)	*p* value	Status
H_1_	Performance expectancy → Trust	0.419	0.004	Supported
H_2_	Effort expectancy → Trust	0.346	<0.001	Supported
H_3_	Hedonic motivation → Trust	-0.079	0.236	Not supported
H_4_	Perceived social presence → Trust	0.187	0.011	Supported
H_5_	Perceived humanness → Trust	0.092	0.822	Not supported
H_6_	Social cognition → Trust	0.512	<0.001	Supported
H_7_	Perceived privacy risk → Trust	-0.565	<0.001	Supported
H_8_	Perceived privacy risk → Perceived privacy concern	0.626	0.002	Supported
H_9_	Perceived privacy concern → Trust	-0.003	0.924	Not supported
H_10_	Trust → Behavioral intention	0.032	0.431	Not supported

### 5.3 Discussion of the findings

The results indicate that out of 10, 6 hypotheses are supported, and the remaining are not supported. Effort expectancy and social cognition both have positive influences on trust. EE represents the functional aspect, while SC the social aspect of the VCED devices. A closer examination into both these constructs reveal that they are related to the perceived competence of the users. This is not only in accordance to the findings of extant research [25], but also confirms the contribution of combining the UTAUT2 with PSR theory.

Perceived social presence also positively affects the overall trust. When there is a feeling of social presence, it creates an engaging environment, which in turn results in creating human-like bonds with machines through the development of mutual understanding and closeness [15]. However, the relatively low weightage of this path is a concern area and indicates that the VCED devices are still in their infancy stage, and not all the users may be comfortable in interacting with them because of their accent [69], misunderstanding keywords, or providing irrelevant information [70]. Under such circumstances, the illusion of interacting with a “*human*” is broken, as it becomes an object of frustration. This also explains as to why the relationship between hedonic motivation and trust is found to be non-significant for the present case. In another study related to the use of voice in autonomous vehicles [71], the authors found that the hedonic aspect acted like a barrier as trust was not fully present. The accent of the VCED devices, their language usage, speed of talking, along with their limited ability to understand human speech are some usability issues that still exists that create barriers in the man-machine relationships.

For the third social factor (perceived humanness), no significant effect was found. This indicates that the users do not find anthropomorphic qualities in the VCED's, and rarely imagine these to be “*real humans*” having emotions. Previous research in [72] has suggested that anthropomorphism depends not only on the presence of human-like attributes, but also on the type of anthropomorphism. Therefore, for explaining such a non-significant result, we would like to refer to the four different anthropomorphic forms that current research considers: structural, gestural, character, and aware [73]. The VCED devices exhibit an aware anthropomorphic form, wherein these devices try to imitate the human capacity for thought, upon the users' interaction. This type of anthropomorphism emphasizes on the common nature of being a human. The VCED devices are supposedly to be intelligent and smart as they interact with their users for information seeking or enjoyment or other utilitarian purposes, thereby being able to actively participate. Although rapid advancements in AI and NLP technologies have helped in this regard, yet user frustrations are common that leaves them unsatisfied [69, 74, 75, 76]. Lack of accuracy, limited semantic understanding capability, lack of service maturity, and limited capability to understand different accents are just some of the limitations that these devices presently have. Consequently, they are not capable of invoking the perceived humanness feeling, due to their lack of maturity and low level of anthropomorphism. Devices having natural language communication capability may lead to weak anthropomorphism (which can occur if people view objects to have some human traits, but do not consider it human as a whole), which may be attributed to the lack of human shape or form (design aesthetics) that the VCED devices lack. Moreover, extant research has found that while a certain level of anthropomorphism increases the users affection, there is a point beyond which anthropomorphism will be not effective [54]. Since the participants of the current study were users experienced with VCED devices, anthropomorphism is less likely to be used as basis for induction, and hence the users do not depend on the humanness aspect to develop a trusted relationship.

In relation to the privacy aspects the results are interesting. Perceived privacy risk has a significant influence on trust [58]. This relationship is negative, indicating that higher the perception of risk, lesser will be the trust on technology. In such a circumstance if risk perception is needed to be reduced, measures should be taken that promote the trusting aspect. Further, the relationship between PPR and PPC is also positive and significant, which is in accordance with current research [58]. It means that people having higher negative privacy perceptions will also have concerns over the voice-based technology, and consequently trust it less. Most surprisingly, the effect of PPC on trust is found to be non-significant, which is completely counter-intuitive [58]. We attribute this finding to the privacy calculus perspective, which states that if people think a technology to be very useful, they will care less about the privacy concerns. AI based technologies that power the VCED devices provide a certain degree of personalization, which in turn triggers a risk-benefit analysis in the users' mind and the privacy concerns become negligible when benefits outweigh the losses. Finally, trust is not found to have any effects on the behavioral intention, which is in sharp contrast to existing findings [61]. The VCED devices are not standalone devices. In fact, they are a central part of any smart-home ecosystem. Many times, there are compatibility issues between VCED devices from multiple manufacturers, due to which they do not work properly. Such frustrations are common among the users, and they have little faith in the inter-working of the devices that translates to a less BI. Another reason as to why trust does not translate to BI maybe because of negative emotions that users have towards the VCED's. They might fear that machines will rule over humans some-day, with humans losing control of the systems. Such feelings have been established in AI based technologies where likeability is replaced by an eerie feeling towards a system beyond a certain limit [54]. Considering that the participants of this study were millennials who generally are tech-savvy, they might have high expectations with regards to the intelligence and anthropomorphism provided by the VCED's, that they are not able to fulfill [11]. Consequently, the trust is not enough that will translate to a higher behavioral intention.

## 6 Theoretical and practical contributions for the CE community

Recently, the advances made in ML, NLP and AI techniques have resulted in the CE community to focus on devices with different modalities of communication, e.g., voice. While the VCED devices open a new era of human-machine communication together with multiple new use-case scenarios, the CE community till date has focused mainly on the engineering aspect of these devices. However, how these devices are perceived by the end users, what are the concern areas, whether the human-like attributes of these devices can ensure a trusted man-machine relationship are some of the grey areas that the CE community needs to investigate. Technological advancements must go hand-in-hand with their diffusion in the society that has been a motto of the CE researchers since the past 60 years. Continuing this tradition there is an imminent need to explore the anthropomorphic aspects of voice-based technology, and how a sociotechnical approach can explore the trust aspect of the users to use these devices. Prior CE literatures as in [12, 13, 34] touch upon this end-user aspect, but in a shallow manner providing only conceptual models and discussions, and not exploring the factors related to trust that can affect the diffusion of this technology in the CE mass market. This is exactly where the current research steps in and analyzes the trust that users develop with the VCED devices from a sociotechnical perspective. The findings have several implications both from a theoretical and practical viewpoint that are discussed next.

### 6.1 Theoretical contributions

In this work an attempt is made to investigate the role of trust and how it affects the behavioral intention to use the VCED devices by using a sociotechnical approach. A research model is developed by integrating UTAUT2, PSR, and privacy- calculus theory. Recent research related to anthropomorphic information systems have repeatedly called for newer ways of evaluating human perception that goes beyond the technical or social approaches currently used, because these systems are unique by having some level of intelligence and human-touch [25, 32, 54]. Current literatures related to anthropomorphic systems are still in its infancy, and mainly conceptual [25]. Moreover, majority of the current studies merely replicate the conventional technology acceptance models like TAM, UTAUT, etc. (Table 1). These works mainly focus on several functional aspects, or even hedonic aspects, but do not consider the unique features of anthropomorphic systems. By adopting a multi-pronged approach including the functional, hedonic and social drivers, we are able to contribute to a more holistic understanding of the acceptance of these technologies.

Second, the findings contribute to the current understanding of trust with the VCED's, and AI based systems in general. The results demonstrate the importance of trust in this aspect, and the importance of the social elements; namely, perceived social presence and social cognition. Previous research in a service-robot context had demonstrated that turn-taking cues lead to developing a positive trusted feeling [37]. The current results expand this knowledge and show that when VCED's are perceived as socially intelligible agents, it leads to developing positive trust. The findings not only contribute to the existing trust literatures, but also enhance the current understanding of social presence and social cognition in relation to interacting with the VCED's and shows that how virtual human-like cues can invoke cognitive competence. This social aspect and its effect on trust has rarely been examined empirically in an human-VCED interaction scenario [14].

Third, the results reveal that privacy concerns do not have any effect on the trust aspect. Specifically, when interacting with voice agents, previous research has shown that users distinguish these devices from their parent companies, i.e. people associate the informational and data loss to the companies and not to the VCED's [77, 78]. This indicates that there are two different sources of privacy and trustworthiness, and during interactions between the users and the VCED's, the effect of privacy concern is extrinsic, which does not affect the trust process with these devices. Although the negative effect of privacy concern on trust is well documented by current literatures, this study finds a different path by which these two aspects intersect that is not determined by the human-VCED interactions. This is a new contribution towards privacy literatures on anthropomorphic systems that highlights the relevance of the roles and social importance that users place on these systems when they are engaging with them, and such relationships place the onus of privacy concerns due to data loss to the manufacturers of these devices.

### 6.2 Suggestions for the CE community

Based on the results a number of practical suggestions are provided. First, the role of hedonic motivations is found to be non-significant in the trust building process. The functional aspects are only related to the utilitarian values. With the growing popularity of the home IoT devices, CE researchers have given considerable efforts to integrate voice capabilities into these to make them more convenient to use for the users. While this improves the utility, however, the problem of interoperability still exists that diminishes such utilitarian effects. A large number of existing consumer IoT solutions are proprietary and designed to work only on specific hardware or infrastructure environment. For example, protocols are tied to specific vendor chipsets or wireless connectivity is bound to a single third-party managed backend. The increasingly connected home IoT environment from an end-user's perspective is close ended, i.e., they are silos that pose multiple problems. Due to such vendor lock-ins, integration of new IoT devices or solutions can create operational issues. There are three ways to tackle this problem: (1) creating and adhering to open industry standards, (2) software-driven technologies, and (3) creating open interfaces. Pertaining to the CE context, the march towards open-source software started long back. In comparison the other two aspects of hardware and networking open-source movement is relatively new, and it is difficult to guess when CE will go “fully open”. The biggest challenge is in terms of hardware that is mostly proprietary. However, we strongly advocate the adoption of an open-source approach that can provide multiple benefits and opportunities to the CE manufacturers, vendors, and end-users. The open-source movement can not only help solving the interoperability issues, but also pave way for new innovations in terms of prototyping, producing, and delivering CE devices.

Second, since both performance and effort expectancies determine the trust, another aspect the CE researchers must keep in mind is about the usability aspect. As per one school of thought an open-source paradigm can adversely affect usability. However, in our opinion open source is the future, and CE quality will not be affected by this, which includes the user experience/usability also. A quicker shift to open source will allow a quicker improvement of many aspects such as security and ergonomics. Product design and aesthetics can impact device usage. Therefore, design and ergonomics is an important consideration too for CE researchers. Specially, in relation to voice, smart speakers are available that usually have the same looks or minor cosmetic changes depending upon the product generation. Since these VCED devices are an integral part of a smart home, they should be offered in various colors, sizes, shapes, and designs that can blend well with the home environment. In fact, previous research has shown that if devices are made that look like humans, it enhances the trusting relationship [25, 54].

Third, the social attributes are another important source of trust building. Accordingly, the focus should be on improving the users trust by developing natural human-like dialogue-based conversational flow by leveraging the benefits of ML and NLP. These technologies have the capabilities to learn not only the users' preferences, but also customize interactions, which will further help to develop the perceptions of social presence and social cognition. In this respect we would like to highlight one significant difference of the current findings from previous findings on anthropomorphic systems is general. With VCED's users tend to develop a weak relationship than other forms of this technology, e.g., social robots. In fact, in the robotics research segment humanness is one very important aspect that researchers focus on, and due to their embodied nature, they can invoke a greater sense of anthropomorphism [54]. However, voice-based anthropomorphism is weak for users to develop a relationship with these. Therefore, focusing on these aspects will help in improving the VCED adoption scenario.

The individuals are concerned about the trustworthiness of these technologies and the companies that produce them. The VCED's need a variety of data from the users to perform effectively, and the users will provide personal information only when they trust the technology and the relevant stakeholders. Thus, the CE community must work in close association with security experts in securing user trust, mainly in relation to the safety of their personal information. This can be done in many ways: by bringing in more transparency in the algorithms and usage of data, clearly communicating the business models with the users and how their data is used, or by providing relevant cybersecurity training to the users. Another challenge specifically for the VCED devices is their lack of a visual user-interface. For example, manufacturers should provide with some type of visual indicators (small screen, LED notifications, etc.) or voice prompts (for e.g., in case of available software updates, or security patches) for notifying the users about the different events. Likewise, when such devices reach their end-of-life or when the users want to dispose them off, these must be equipped with a master-reset button that will ensure a complete data wipe from their storage. Additionally, as the VCED devices provide personalized service they gather a lot of data, which makes it very important to establish strong privacy norms and regulations by the government to protect user's data and rights.

Lastly, the importance of privacy cannot be underestimated. Consequently, in this respect we recommend adhering to standard privacy frameworks from organizations like NIST and ISO while designing the products. Privacy by Design (PbD) is a related concept in this aspect that should be taken into consideration. By adhering to the PbD principles the CE manufacturers can be more accountable to the data they collect from the users, and whether the data is being used in a fair manner. By invoking transparency and a fair usage policy of user data the CE manufacturers can promote confidence among the users, which in turn will improve the trust level. A still greater challenge in this respect is the variation of privacy perceptions in different countries based on culture. Although PbD is an important design philosophy, yet this concept should be broadened to include cultural variations too, signaling a transformation from the traditional PbD to a Privacy by Culture (PbC) scenario. While PbD will help in improving the aspects related to data transparency, the country specific variations can be dealt with in terms of PbC. The PbC concept can be incorporated by having specific software for different countries that can handle the variations. Taking these steps will result in gaining more user confidence and improve the trust aspect.

## 7 Limitations and future work

This work is not without limitations. First, the research is conducted in India, and Thailand i.e., in an Asian context. Therefore, the results are representative of an Eastern collectivist society, and may not be applicable in the Western individualistic context. With current literatures continuously debating the relevance of factors according to the cultural context, it will be best if future research is conducted from a wider demographic perspective that will involve greater cultural variations. Second, the research data was collected from millennial generation users. These are young people who are tech-savvy, and normally early adopters of technology. Therefore, they are different from other groups like the late adopters, or those who do not have any experience using this technology. Although, we focus on this group and believe that using such a sample better represents the trust building process with the VCED's, future studies can investigate the other user groups like late adopters, and non-tech savvy users. Third, the trusting beliefs in this work are explored post device usage. However, as a part of future work it will be interesting to do a time-series analysis, exploring right from initial trust to trust after device usage, and see if there is any difference between these two. Confirmation of pre-expectations and post-expectations will help the CE research community to understand those factors that must be given preferences while designing these voice-based systems. Finally, the effect of moderating variables like gender or usage experience was not considered in this work. Future studies can focus on these aspects and explore their roles. Likewise, newer techniques like FsQCA (Fuzzy-set Qualitative Comparative Analysis) may be used for the purpose of data analysis.

## Declarations

### Author contribution statement

Debajyoti Pal: Conceived and designed the experiments; Performed the experiments; Analyzed and interpreted the data; Wrote the paper.

Pranab Roy: Conceived and designed the experiments; Performed the experiments; Analyzed and interpreted the data.

Chonlameth Arpnikanondt: Conceived and designed the experiments; Contributed reagents, materials, analysis tools or data.

Himanshu Thapliyal: Analyzed and interpreted the data; Wrote the paper.

### Funding statement

D. Pal was supported by the 10.13039/100007684Asahi Glass Foundation.

### Data availability statement

Data will be made available on request.

### Declaration of interests statement

The authors declare no conflict of interest.

### Additional information

No additional information is available for this paper.
